# Evaluation of a memory and psychomotor training for cancer patients with cancer-related cognitive impairment: a study protocol for a prospective randomized controlled single-center trial in Germany

**DOI:** 10.1186/s13063-025-09211-z

**Published:** 2025-10-27

**Authors:** Karin Meng, Joschua M. Reis, Adele Kruse, Isabell Lee, Anna Ballweg, Mia Wallis, Viktoria Rücker, Peter U. Heuschmann, Elisabeth Jentschke

**Affiliations:** 1 Comprehensive Cancer Center Mainfranken, University Hospital Würzburg, Josef-Schneider Str. 6, Haus C16, Würzburg, 97080 Germany; 2 Institute of Clinical Epidemiology and Biometry, University of Würzburg, Am Schwarzenberg 15, Haus A15, Würzburg, 97078 Germany

**Keywords:** Cancer, Psycho-oncology, Cancer-related cognitive impairment, Memory training, Physical training, Cancer-related fatigue

## Abstract

**Background:**

Cancer-related cognitive impairment (CRCI) is common and can affect the quality of life for cancer patients. Nonpharmacological interventions as cognitive training or exercise are recommended. However, research that includes both self-reported and objective outcome measures, along with longer-term follow-up to identify the most effective interventions, is lacking. This trial aims to evaluate a combined memory and psychomotor training compared to usual care with regard to objective and subjective cognitive function for cancer patients with curative treatment and CRCI.

**Methods:**

The study is a single-center, prospective, open-label, 1:1 randomized controlled superiority trial. 170 eligible patients with (i) a curative tumor diagnosis, (ii) after chemotherapy, radiation therapy, or ongoing antibody therapy, and (iii) self-reported CRCI are recruited by study team members at a German university hospital (first planned enrollment: July 2023). Participants are randomly allocated to the intervention group (IG) or the waiting control group (CG). The IG receives a standardized interactive online group memory and psychomotor intervention (8 sessions of 60 min once a week). The CG receives usual care treatment. Participants complete assessments at baseline (t1), after the intervention (t2 IG; post-intervention) or 3 months after baseline (t2 CG), and 6 months after baseline (t3; follow-up). The primary outcome is the alertness assessed with a computer-based test (Test Battery of Attentional Performance (TAP); scale intrinsic alertness) in short- and intermediate-term (t2, t3). Secondary outcomes include objective and self-reported cognitive function, symptoms of cancer-related fatigue, quality of life, symptoms of depression, symptoms of anxiety, and physical performance. Outcomes are assessed using neuropsychological tests, validated questionnaires, and a physical function test. Training adherence and treatment satisfaction are evaluated using a self-developed questionnaire. Assessors are blind at t1; care providers are blinded. Harms are documented by study team members. Intervention effects will be evaluated separately for each follow-up time point using analysis of covariance (ANCOVA), adjusting for baseline values. Furthermore, subgroup-related treatment effects will be explored.

**Discussion:**

Overall, the results will enhance our understanding of the effectiveness of the intervention in both subjective and objective cognitive outcomes in cancer patients experiencing subjective cognitive impairment. If the training shows positive effects, it should be implemented in psycho-oncological routine care.

**Trial registration:**

German Clinical Trials Register DRKS00027361. Registered on 2nd December 2021.

**Supplementary Information:**

The online version contains supplementary material available at 10.1186/s13063-025-09211-z.

## Background

Patients with non-central nervous cancer frequently report cognitive changes and impairment, a condition known as cancer-related cognitive impairment (CRCI). Common symptoms include declines in short-term and working memory, attention, executive functions, and information processing speed [1, 2]. The development of CRCI seems to be multifactorial. However, the exact mechanisms underlying these cognitive changes are not completely understood. Assumed risk factors include cancer, administered treatment, genetic predisposition, age, comorbidities, and psychological factors such as anxiety, depression, or fatigue. Multiple mechanisms such as neuroinflammation, direct neurotoxic injury, endothelial dysfunction, or hormone changes are discussed [2, 3]. Additionally, a characteristic of cancer-related fatigue (CrF) is cognitive tiredness that is disproportionate to activity and usual functioning, which is referred to as the cognitive aspect of fatigue (NCCN Guidelines CrF V2.2023; [4]). These cognitive issues can negatively impact daily living activities, social participation, and overall quality of life during cancer treatment and survivorship [1, 2]. As a result, there is increasing interest in potential treatments.

The globally reported prevalence of cognitive impairment in cancer patients varies widely, ranging from 10 to 83% [2, 5–7]. This discrepancy may stem from differences in cancer populations, definitions, and cognitive assessment tools, such as self-report questionnaires versus neuropsychological tests (e.g., [6–8]). The Functional Assessment of Cancer Therapy Cognitive Function (FACT-Cog) is the most common patient-reported outcome questionnaire, while neuropsychological tests are more heterogeneous [6] despite existing recommendations [9]. Generally, self-reported cognitive complaints are higher than impairments determined in neuropsychological tests (e.g., [7]). This discrepancy can be attributed to psychological factors, such as anxiety, depression, fatigue, and insomnia, which may have a greater negative impact on subjective cognitive issues than performance on objective tests. Additionally, compensatory activation of brain regions may help maintain performance in neuropsychological assessments [2].

Nonpharmacological interventions are recommended for treating CRCI [2, 3, 5]. Cognitive rehabilitation programs (CR; [10, 11]), which include cognitive training (CT), cognitive behavioral therapy (CBT), and psychoeducation as well as meditation/mindfulness-based interventions (MBSR; [12]), might improve both subjective or objective cognitive impairment [13, 14]. Evidence supporting exercise training’s benefits is limited to self-reported outcomes [15]. A network meta-analysis on nonpharmacological interventions for CRCI indicates the best effects on subjective cognitive function for CT, CR, meditation/MBSR, and exercise interventions [16]. Another network meta-analysis focusing on neuropsychological interventions reports the best effects for CBT, CR, and CT in terms of subjective cognition. CT was the most effective intervention for managing verbal memory, meditation/MBSR for enhancing attention, psychoeducation for managing executive function, and supportive care for processing speed [17].

However, these findings are limited by the heterogeneous nature of the included studies, i.e., variations in cancer type, time of intervention, types of interventions used, and measurement methods [13, 18]. Furthermore, many studies lack measurements of objective cognitive performance. Therefore, further research that includes both self-reported and objective outcome measures, along with long-term follow-up, is needed to identify the most effective interventions.

In older adults (75 years or older) a combined cognitive and physical training approach, referred to as psychomotor training, showed significant positive effects on cognitive function, cognitive impairment, depressive symptoms, health status, and well-being lasting for up to 5 years post-training (SimA; [19]). Psychomotor training is defined as the integration of mental perception and behavior with motor responses. Movement is viewed as a developmental stimulus that promotes perception and behavior, along with related planning and thinking processes. However, to date, no comparable intervention has been evaluated in cancer patients experiencing CRCI.

### Objectives

Our study aims to prove the effects of a combined memory and psychomotor intervention (MPMI *M*emory and *P*sycho*M*otor *I*ntervention) compared to usual care for cancer patients with curative treatment and CRCI.

#### Main research questions

- Does the memory and psychomotor training for cancer patients with CRCI has an effect on the alertness (primary outcome) compared to a usual care waiting group?
- Does the memory and psychomotor training for cancer patients with CRCI has an effect on objective and subjective cognitive functions, symptoms of cancer-related fatigue, quality of life, symptoms of depression, and anxiety (secondary outcomes) compared to a usual care waiting group?

We hypothesize that the MPMI has a positive effect on alertness (primary hypothesis). In addition, we expect superior efficacy of the intervention regarding cognitive performance, symptoms of cancer-related fatigue, quality of life, symptoms of depression, and anxiety symptoms.

As *secondary research questions*, we explore moderator effects of disease-related and socio-demographic parameters. Furthermore, we investigate if the intervention affects physical performance and whether the perceived cognitive changes correspond to the cognitive performance test.

## Methods/design

### Trial design

The study is a single-center prospective, randomized controlled trial (RCT) with three measurement times. Cancer patients with CRCI are randomly assigned to the MPMI group (IG) and the waiting control group (CG). The IG receives the memory and psychomotor training, whereas the CG receives usual care treatment. Participants complete assessments at baseline (t1), after the intervention (t2 IG; post-intervention) or 3 months after baseline (t2 CG), and 6 months after baseline (t3; follow-up). Participants of the CG can participate in the training after the last follow-up assessment.

### Study setting and participants

The study is performed at the University Hospital Würzburg, Comprehensive Cancer Center Mainfranken (CCCMF), Germany. The university hospital is a certified cancer center for the types of tumors included.

The inclusion criteria are (i) a curative tumor diagnosis (breast cancer, blood cancer, gastrointestinal tumors, malignant melanoma), (ii) after chemotherapy, radiation therapy, after or ongoing antibody therapy, (iii) age between 18 and 79 years, (iv) and self-reported cognitive impairment with onset temporally related to cancer diagnosis (FACT-COG PCI18, cut-off score ≤ 55 [20, 21]).

The exclusion criteria are a lack of German language abilities or language disorders, severe cognitive impairment such as dementia, brain tumors/central nervous system cancer, immobility or non-resilience of the patient, and a palliative state.

Furthermore, eligible patients must have access to a terminal device with Internet access and must provide written informed consent. Assessment of exclusion criteria is detailed in Supplementary material 1.

### Intervention

#### IG

Patients in the intervention condition receive a standardized online memory and psychomotor training (MPMI). The training is accompanied by a workbook for home practice and an educational booklet comprising a summary of each session.

The intervention consists of eight interactive online training sessions of 60 min each that are provided in a group of eight to twelve participants once a week. Two trained scientists conduct the training following a written manual implemented in a PowerPoint presentation. A psychologist administers memory training, and a sports scientist instructs psychomotor exercises. Sessions take place using the video communication application Zoom (Zoom Video Communications, San Jose, CA).

Each session follows the same structure and consists of three parts: (1) Introduction with feedback on home practice, a mood scale, and warm-up exercises (10–15 min). (2) The main part comprises psychoeducation, memory, and psychomotor exercises (30–40 min). (3) The final part includes relaxation, mood scale, summary, and home practice instructions (10–15 min). Feedback is always discussed with all participants. The mood scale is assessed individually. Participants are free to share their experiences and mood, or can keep them to themselves. Table 1 summarizes the psychoeducational content and cognitive and psychomotor exercises.

**Table 1:** Content of the online MPMI

Module	Psychoeducation	Cognitive training	Psychomotor training
1	Attention and concentration	Color-Word-Test (Stroop-Test)	Breathing exercise, juggling, relaxation—palming
2	Multi-store model part 1: Perception processing, sensory register, mindfulness	Verbal training: name chain, Auditory perception exercise, mind/mental walk	Finger counting exercise, juggling, relaxation—posture exercise
3	Multi-store model part 2: Short-term store	Verbal training: increasing adjectives with neologism, traffic sign recall, anagram	Finger-tapping, juggling, rope-sensation, relaxation—posture/breathing
4	Multi-store model part 3: Long-term store, mnemonic strategies	Visualization techniques: memorizing names by associative mnemonic image, story method	Ball balancing, juggling, 4–6–8-breathing technique
5	Multi-store model part 4: Retrieval strategies	Verbal training: expressions with color names, alphabet pegs, chunking by categorization, repetition with rhythm	Diagonal motion exercises, palming, yoga
6	Daily reminders, automatisms and routines, SQ3R-Method	Active describing: memorizing a picture by describing with own words	Swing double ball rod, juggling, relaxation—posture exercise
7	External and internal memory aids: checklists, method of loci	Making a checklist: travel preparations, loci technique exercise	Sequence dance, throw chain, progressive muscle relaxation
8	Repetition of modules 1–7	Movement based learning: “packing a suitcase”, story method, ambiguous images, cities and sights	Movement based learning: “packing a suitcase”, juggling

The detailed trainer manual is available in German and can be requested from the corresponding author

The MPMI program was based on existing concepts, especially the SimA intervention [22, 23]. Dr. E. Jentschke (neuropsychologist, SimA trainer for memory/psychomotor) supervised the conception of the memory training by the study team. The psychomotor exercises united the experience of movement, action, feelings, and perception through senses, thinking, and communication [24]. The proportion of the psychomotor and memory training is equivalent to the ratio of the combined training groups in the SimA study [19]. However, the MPMI has a reduced intensity (time, duration). The sessions are shorter (60 min instead of 135 min) due to the online format and the fatigue symptoms of the participants. Furthermore, it requires less time and effort (once a week for 8 weeks instead of twice a week for 10 weeks duration).

To foster treatment adherence and prevent overload, participants can take an individual break at any time. Examples of short screen breaks are shown during the relaxation sessions (e.g., palming). In addition, active participation during the training is voluntary, and participants can choose their level of difficulty for each psychomotor exercise. To foster home practice, barriers and achievements in homework are discussed at the beginning, and a question and feedback round is administered at the end of each session. Exchange on these aspects takes place in the whole group, respectively. However, not all participants are required to contribute. Attendance at the individual sessions is documented. In case of absence without prior notice, participants are contacted to address possible reasons. However, participants can discontinue the intervention at any time.

To ensure treatment integrity in the training manual, an additional research assistant regularly observes and documents the training units by using a standardized observation protocol.

#### CG

Patients of the CG receive usual care and can use offers of supportive care at the CCCMF (e.g., psycho-oncological, exercise, and dietary counselling, relaxation groups, and complementary medicine consultation). They can participate in the MPMI after the last follow-up assessment. This control condition was chosen for ethical reasons.

There is no restriction for other treatments in both groups.

### Outcomes and measurements

Table 2 presents an overview of outcomes and assessments. The primary outcome is the response time in milliseconds of the intrinsic alertness assessed with the computer-based Test Battery of Attentional Performance (TAP version 2.3.1; PSYTESTS; [25, 26]). Secondary outcomes are objective and self-reported cognitive function, symptoms of cancer-related fatigue, quality of life, symptoms of depression, symptoms of anxiety, and physical performance. All outcomes are assessed using neuropsychological tests, validated questionnaires, and a physical function test. Furthermore, training adherence and treatment satisfaction are evaluated using a self-developed questionnaire based on further studies (e.g., [27, 28]). In addition, sociodemographic and medical data are collected by self-report or by the medical record.

**Table 2:** Outcomes, measures, and assessment

		**Time of completion**	**Assessment**		
**Outcomes**	**Measures**	**(minutes)**	**t1**	**t2**	**t3**
***Cognitive functioning***	***Tests***				
Alertness (primary)	Test of Attentional Performance TAP – subtest alertness [25, 26]	5	x	x	x
Go/Nogo	Test of Attentional Performance TAP – subtest Go/Nogo [25, 26]	2	x	x	x
Working memory	Test of Attentional Performance TAP – subtest working memory [25, 26]	5	x	x	x
Memory: short term, long term, recognition	Verbal learning and memory test VLMT [29]	25	x	x	x
Speed of information processing	Trail making test part A TMT A [30]	2	x	x	x
Divided attention, cognitive flexibility	Trail making test part B TMT B [30]	5	x	x	x
***Physical functioning***	6-min walk test 6MWT [31]	6	x	x	x
***Subjective health***	***Questionnaires***				
Cognitive function	Functional Assessment of Cancer Therapy—Cognition, FACT-Cog (version 3) [32]	10	x	x	x
Cancer-related fatigue	European Organization of Research and Treatment of Cancer Quality of Life Questionnaire-Fatigue 12 questionnaire EORTC QLQ-FA12 [4]	5	x	x	x
Health-related quality of life	European Organization for Research and Treatment of Cancer quality of life questionnaire EORTC QLQ-C30 [33]	10	x	x	x
Depression	Patient Health Questionnaire PHQ-9 [34, 35]	5	x	x	x
Anxiety	The Generalized Anxiety Disorder-7 questionnaire GAD 7 [36]	5	x	x	x
Training adherence	Self-developed questionnaire	5	–-	x	x
Treatment satisfaction	Self-developed questionnaire	5	–-	x	–-

t1 indicates before intervention; t2, after 3 months; t3, after 6 months. To objectify cognitive performance decline (cognitive fatigability), the TAP subtest alertness was repeated at the end of the test battery to measure

#### Primary and secondary outcome measure

##### TAP [25, 26]

The TAP provides a computer-based battery of different tests that can be used to assess the various aspects of attention in a differentiated way. Detailed information is available at https://www.psytest.net/en/test-batteries/tap/subtests. Norm values (i.e., age-, gender-, and education-corrected T-scores) are provided for all subtests. These are used for the analyses.

##### TAP—alertness

Alertness refers to the condition of general wakefulness that enables a person to respond quickly and appropriately to any given demand. It is the pre-requisite for effective behavior, and is in this respect the basis of every attention performance [25, 26]. The test examines reaction time under two conditions: In the first condition, intrinsic alertness (primary outcome) is measured by a visual stimulus that appears on the monitor at a randomly varying interval. The subject should respond as quickly as possible by pressing a key (“tonic arousal”). In the second condition, “phasic arousal” or temporal orientation of attentional focus is measured in response to the same visual stimulus preceded by a cue stimulus presented as a warning tone. Reaction time in milliseconds (mean, standard deviation (SD)) and norm-values are available, respectively.

##### TAP—Go/Nogo

The task tests a form of behavioral control, i.e., the ability to perform an appropriate reaction under time pressure and to simultaneously inhibit an inappropriate behavioral response [25, 26]. The subject has to respond to predictably occurring stimuli that require a selective reaction, that is, to react or not to react. Test form “1 of 2” (1 of the 2 stimuli is critical) is administered. Norm-values for reaction time and for mistakes are provided.

##### TAP—working memory

The task examines the control of information flow and the updating of information in working memory [25, 26]. A sequence of numbers is presented to the subject on the monitor. The subject is required to determine whether each number—out of a sequence of numbers presented on the monitor—corresponds with the previous number. The Level 1 test condition is being carried out. Norm values are available.

##### Verbal learning and memory test (VLMT; [29])

The VLMT represents the German Version of the Auditory Verbal Learning Test (AVLT; [37]), which was translated and evaluated in a German population. The test measures verbal memory and learning ability with several scores: learning, post-interference recall, delayed recall, and delayed recognition. Parallel versions of the word lists are used for repeated testing. Norm values are available, including age-corrected *T*-scores for most age groups. *T*-scores derived from the total normative sample are used for the delayed recall score.

##### Trail making test (TMT; [30])

The TMT consists of two parts (A, B) that examine the speed of information processing and divided attention/cognitive flexibility. TMT-A requires the person to draw lines sequentially connecting 25 encircled numbers distributed on a sheet of paper. Task requirements are similar for TMT-B, which requires the subject to draw lines alternating between ascending numbers and letters. The score on each part represents the amount of time required to complete the task. Therefore, lower scores correspond to better cognitive function.

##### Functional Assessment of Cancer Therapy—Cognitive Function (FACT-Cog; [32])

The questionnaire (version 3) assesses subjective cognitive impairment with 37 items rated on a 5-point Likert-type scale (0 = never/not et al.; 4 = several times a day/very much). Standard scoring comprises four scales: perceived cognitive impairment (PCI; 18 items, score range: 0–72), impact of perceived cognitive impairment on quality of life (CogQOL; 4 items, score range: 0–16), comments from others (CogOth; 4 items, range: 0–16), and perceived cognitive abilities (CogPCA; 7 items, range: 0–28). Higher scores correspond to a better subjective cognitive function.

##### EORTC Cancer Related Fatigue Module (EORTC QLQ-FA12; [4])

Cancer-related fatigue is assessed with the supplementary fatigue scale of the European Organization for Research and Treatment of Cancer (EORTC) Quality of Life Questionnaire. The questionnaire comprises twelve items of fatigue’s physical (e.g., lacked energy, feel sleepy during the day, etc.), cognitive (e.g., feel confused, have trouble thinking clearly, etc.), and emotional aspects (e.g., feel helpless, frustrated, etc.), rated on a 4-point Likert scale (1 = not at all; 2 = a little; 3 = quite a bit; 4 = very much). The total score is transformed to a scale ranging from 0 to 100, with higher scores representing more severe fatigue symptoms [4, 38].

##### EORTC quality of life questionnaire (EORTC QLQ-C30; [33])

Patients complete the validated European Organization for Research and Treatment of Cancer Quality of Life Questionnaire Core 30 (version 3.0). This 30-item questionnaire comprises five functional subscales, i.e., physical, role, cognitive, emotional, and social functioning, a global QoL subscale, as well as nine symptom scales or items. Subscale scores are transformed into a range from 0 to 100, with higher scores indicating higher functioning and QoL or higher symptom burden.

##### Patient Health Questionnaire-9 (PHQ-9; [35])

In order to assess depressive symptoms, we use the PHQ-9, a short questionnaire which evidenced high reliability and validity. Patients rate nine items that refer to symptoms of a depressive episode according to DSM-IV criteria (e.g., depressed mood, anhedonia) during the last 2 weeks on a 4-point Likert scale (0 = not at all; 1 = several days; 2 = more than half the days; 3 = nearly every day). Items are combined into a sum score (range: 0–27), with higher scores indicating more severe depressive symptoms. Cut-off points of 5, 10, 15, and 20 can be interpreted as representing mild, moderate, moderately severe, and severe depression, respectively [34]. The sum score is used for the analysis.

##### Generalized Anxiety Disorder scale (GAD-7; [36])

The GAD-7, a questionnaire with high validity and reliability, is used to measure anxiety symptoms. The questionnaire consists of seven items that address core symptoms of generalized anxiety disorders during the last 2 weeks (e.g., uncontrollable worries, nervousness) according to DSM-IV criteria. The items are answered on a 4-point Likert scale (0 = not at all; 1 = several days; 2 = more than half the days; 3 = nearly every day). Items are summed up resulting in an anxiety score ranging from 0 to 21, with higher scores indicating a higher expression of anxiety symptoms. Scores of 5, 10, and 15 represent mild, moderate, and severe levels of anxiety, respectively. For analysis, the sum value is applied.

#### Other outcome measure

##### 6-min walk test (6MWT)

We use the 6MWT to assess the functional capacity [31]. The patient must walk for 6 min over an incline-free circuit or a walkway of at least 30 m in length. The goal for the patient is to achieve as much distance as possible in the given time. The total distance walked is measured in meters. Norms are available [39].

##### Training adherence

The frequency of addressed practice behavior during the last month before assessment is recorded. Participants report how often per week and how long per session (minutes) they perform memory or relaxation/mindfulness exercises. Two training scores (in minutes per week) are calculated by multiplying the number of sessions by minutes per session. In addition, they are asked how often per week and how long per session they perform strenuous, moderate, and light physical exercise outside of work duties (adapted version of the Godin Leisure-Time Exercise Questionnaire; [40]). A total physical activity score (in minutes per week) is calculated by multiplying the total number of sessions per week in each domain by the minutes per session in each domain. For each of the scores, higher scores indicate a higher level of training adherence.

##### Treatment satisfaction

Participants are asked to judge the MPMI using 22 items. Patients were asked to judge the structure (e.g., duration, group size, online setting; 4 items) and implementation (e.g., content, exchange of experience, booklet; 6 items) on a 6-point scale (1 = very good; 6 = insufficient). Furthermore, the usefulness of the program is rated with four items (i.e., theoretical content, group interaction, psychomotor exercises, and memory exercises) on a 4-point scale (1 = is completely true; 4 = does not apply at all). In addition, two items inquire about training performance (memory exercises, psychomotor exercises) outside the sessions on a 5-point scale ranging from “daily” (1) to “never” (5). Furthermore, four items address the general satisfaction with the program (e.g., recommending the training to others) on a 4-point scale (1 = absolutely; 4 = in no case). Lower scores correspond to higher satisfaction and training performance, respectively. Finally, participants can give feedback on the most helpful aspects and make suggestions using two items with an open response format.

### Participant timeline

**Fig. 1:**
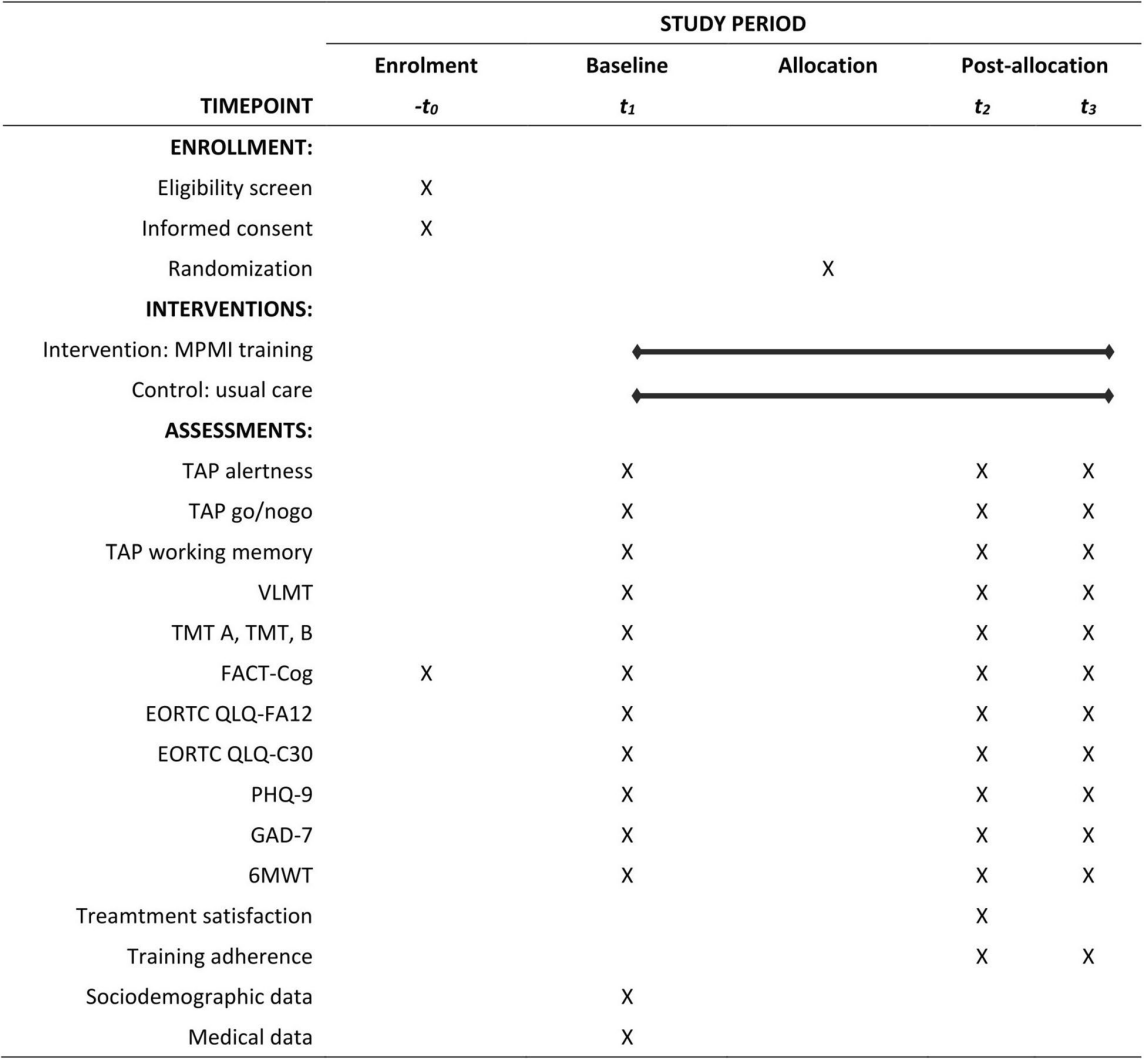
SPIRIT schedule of enrollment, interventions, and assessments

Figure 1 presents the SPIRIT schedule of enrollment process, intervention, and assessments performed on participants.

### Sample size

The primary outcome is the change in the alertness as reaction time in milliseconds (without critical stimulus/warning signal) during the intervention (t1–t2). Based on data from a neuropsychological training in a rehab setting [41], a mean difference of 16 ms (SD = 46.5) between the intervention and control group was estimated.

Therefore, sample size was powered to detect a significant difference of 16 ms (SD = 46.5) in the primary outcome between the study groups (2-sided* α* = 0.05, 1−*β* = 0.80) using analysis of covariance (ANCOVA) and adjusting for baseline values. We assumed a *R*^2^ of 0.5 for the regression of the response on only the baseline covariate. Thus, a total of *n* = 136 persons (*n* = 68 per group) is required. We will include *n* = 170 patients (*n* = 85 per group) based on an estimated dropout rate of 20%.

Furthermore, medium between-group effects (*d* = 0.48) on secondary outcomes can be detected (*p* < 0.05, 1− *β* = 0.80). As power to detect smaller between-group effects on secondary outcomes may be lacking, effect sizes and accompanying 95% confidence intervals will be reported throughout. Sample size calculation was conducted in Power Analysis & Sample Size PASS 2020 (NCSS, LLC, Kaysville, Utah; https://www.ncss.com/software/pass) by a statistician from the Institute of Clinical Epidemiology, University of Würzburg.

### Recruitment

Recruitment is based on the ONKOSTAR tumor documentation system (T-Choice software). A filter is applied to identify people between the ages of 18 and 80 years with an eligible diagnosis. Subsequently, a researcher checks the inclusion and exclusion criteria in the electronic patient file. After screening, potential participants are contacted and informed about the study via phone. Persons interested in the study receive the study information by mail. After signing the informed consent form and completing the FACT-Cog online, participants with a PCI18 score of ≤ 55 (cut-off value representing self-reported cognitive impairment) are included.

In addition to the electronically selected patients, members of the psycho-oncological team of the CCCMF can recommend patients for the study. Furthermore, interested patients can contact the MPMI team after being drawn to attention by MPMI flyers distributed at the oncological departments and the hospital homepage.

## Assignment of interventions

### Randomization and allocation concealment

All participants are randomly assigned to the IG or CG after consent is given and initial assessment and questionnaires (t1) are completed. A block randomization procedure (block size: 2 and 4) with an allocation ratio of 1:1 is used. A member of the Institute of Clinical Epidemiology and Biometry at the University of Würzburg (external randomization) created for each of the eight-session groups a separate randomization list with computer-generated random numbers, which is stored locked at the IKE-B.

Members of the research team are informed about the allocation after the baseline assessment. Therefore, a consecutive list with the participants’ IDs (research codes) of each session group is sent per e-mail to the IKE-B member. Randomization results are added to the IDs and sent back per e-mail afterwards.

### Blinding

Blinding of participants and therapists is impossible due to the nature of the intervention (active training group) and waiting control condition. Further care providers are blind to study participation and/or assignment. Furthermore, assessors are blind to treatment allocation at baseline assessment. For organizational reasons, they cannot be blinded by follow-up assessments. Two clinical research associates must take on several tasks and are responsible for enrollment, data collection and management, and intervention implementation.

A research assistant at the Institute of Clinical Epidemiology and Biometry at the University of Würzburg will perform statistical analysis. Therefore, an external researcher can analyze data and prove intervention effects (main research question).

## Data collection, data management, and confidentiality

After inclusion in the study, medical data are extracted from the electronic patient file. Outcomes and other parameters are assessed using online questionnaires and cognitive and functional performance tests at t1 to t3. The assessments are conducted in the CCCMF using a standardized case report form (CRF). Participants receive a link to the online questionnaires 1 week before the data collection appointment in the clinic. These can be completed within 6 days via an online platform (Evasys GmbH). Researchers contact non-responders by e-mail or phone to ensure completion.

All data are transferred to Microsoft Excel (2016) and stored on secured servers with regular backup. Test sheets are stored in locked cabinets. Only the members of the research team have access to the data. Double data entries are processed for 20% of the manually transferred data (test data, medical data) to assess data quality. Moreover, range checks for data values are performed.

All data are pseudonymized to ensure confidentiality. For this purpose, each study participant is assigned a research code. The password-protected code list (including name, date of birth, SAP research number, and contact data) is stored locked on a server and file folder that is inaccessible to the public. It is accessible only to the research members responsible for the assignment and data collection.

The principal investigator will retain all collected data for at least 10 years. At the end of the study, the code list will be deleted.

### Statistical analysis

Statistical analysis will be performed using R 4.5.1 (R Foundation; https://www.r-project.org/), SAS 9.4 (SAS Campus Drive, Cary, NC, USA), and SPSS (IBM Corp, Armonk, NY, USA) version 29 Statistics for Windows. Depending on missing data analysis, missing data for outcome variables in accordance with missing completely at random (MCAR) or missing at random (MAR) assumptions will be imputed using a multiple imputation procedure. In addition, missing values due to drop-out or data not missing at random (NMAR) will be analyzed by pair-wise deletion. Drop-out analyses will be carried out by independent group comparisons using t-tests for continuous variables and chi-square tests for categorical variables. Descriptive analyses will be performed concerning socio-demographic and medical data.

#### Main research question

The primary and secondary endpoints will be analyzed according to the intention-to-treat principle. Treatment effects (between-group effects) will be calculated separately for each follow-up time point using analysis of covariance (ANCOVA) adjusting for baseline values. Statistical significance (*p* < 0.05, two-sided), difference in means with 95% confidence intervals (CI), and effect sizes (ή^2^) will be reported for all outcomes. Furthermore, within-group effects, including standardized effect sizes (SES) and accompanying 95% CI, will be calculated for both study groups. Bonferroni corrections to adjust the significance level for multiple tests are planned within secondary cognitive outcomes. Further secondary outcomes will be analyzed exploratorily without adjustment. A sensitivity analysis is conducted for primary analysis by additionally including the group as random effects in a linear mixed model for the primary endpoint to estimate the effect of the group.

#### Secondary research question

Moderator analysis will be performed by including the moderator variable as an additional factor or covariate and examining interaction effects. Significance levels for interaction effects will not be adjusted for multiple tests due to their exploratory nature.

We will also use ANCOVA adjusting for baseline values to examine the effect on physical performance.

Furthermore, correlation- and regression analyses between subjective cognitive changes and the changes in cognitive performance tests will be conducted.

### Monitoring

No data monitoring committee (DMC), interim analyses, and auditing were planned. The study team consists of two independent working groups (including biometric expertise) that are committed to ethical standards and the guidelines of “Good research practice”. The study results are reported to the ethics committee. There is no need for a DMC due to short duration, minimal risks, and no treatment restrictions.

Regular meetings of the team members and the principal investigator are implemented to discuss recruitment, assessment, data management, and intervention implementation.

Furthermore, ethical issues are monitored. The risks of harm to participants are minor. However, when dealing with cognitive impairment, cognitive assessment and training may lead to psychological stress. If participants discontinue the assessment or intervention, the reasons (e.g., adverse effects on psychological well-being) are documented by the study team members. A serious adverse event (SAE) is suicidality and suicide, which may or may not be causally related to study participation. SAEs will also be documented and immediately reported to the principal investigator.

### Ethics, consent and permissions

The study conformed to the principles of the Declaration of Helsinki. The Ethics Committee of the University of Würzburg approved the study protocol on 10.11.2021 (reference number: 173/21). All changes to the study protocol will be submitted to the Ethics Committee for approval. Participation in the study is voluntary and based on written informed consent. Eligible participants will be informed about all relevant aspects of the study, in particular, that participation in the study is voluntary and that they can withdraw their consent at any time without incurring any disadvantages.

The study team members will publish this study protocol and study results; there are no publication restrictions.

#### Patient or public involvement

Patients were not involved in the development of the study. However, feedback on the group program will be collected during the study. Furthermore, after the trial concludes, lay-language summaries will be disseminated to participants to ensure they are informed of the results. The study results will also be presented at informational events for patients. Patient perspective will be used to revise the MPMI before dissemination.

## Discussion

Cancer-related cognitive problems can significantly impact the quality of life for individuals undergoing cancer treatment and during survivorship. Non-pharmacological interventions that have shown benefits in addressing subjective or objective cognitive impairment include CT, CBT, meditation/MBSR, and exercise training. However, more research is needed to explore various cancer types, the timing of interventions, self-reported and objective outcome measures, and long-term follow-up to determine the most effective interventions and identify the patient subgroups that benefit the most.

In this RCT, we will evaluate the short- to intermediate-term effectiveness of a combined online memory and psychomotor intervention compared to usual care for cancer patients experiencing subjective cognitive impairment. Additionally, we will explore treatment effects related to specific subgroups.

Methodological challenges may arise due to the online training format, training adherence, the extensive data collection at three measurement appointments over 6 months, and the need to account for multiple potential confounders.

Overall, the results of this study will enhance our understanding of the effectiveness of a combined online memory and psychomotor intervention in both subjective and objective cognitive outcomes. If the MPMI shows positive effects, it should be implemented in psycho-oncological routine care.

## Trial status

This trial is at protocol version 6, dated 28.07.23. Date of first enrollment: 19.12.2023. Recruitment will be completed at the end of June 2025.

## Supplementary Information

Supplementary Material 1.

## Data Availability

Only the research team will have access to the final data set and statistical code; there are no data sharing plans. However, this study protocol will be published and made publicly available. In addition, the trial protocol and further materials (in German) will be available upon reasonable request by contacting the corresponding author.

## Abbreviations

6MWT: 6-Minute walk test
CCCMF: Comprehensive Cancer Center Mainfranken
CG: Waiting control group
CRCI: Cancer-related cognitive impairment
CrF: Cancer-related fatigue
CRF: Case report form
DSM-IV: Diagnostic and Statistical Manual of Mental Disorders
EORTC: European Organization For Research and Treatment of Cancer Quality of Life Questionnaire
FACT-Cog: Functional Assessment of Cancer Therapy—Cognitive Function
GAD-7: Generalized Anxiety Disorder scale
IG: Intervention group
MPMI: Memory and psychomotor intervention
PCI: Perceived cognitive impairment
PHQ-9: Patient Health Questionnaire-9
QLQ-C30: Quality of Life Questionnaire Core 30
QLQ-FA12: Quality of Life Core Function Questionnaire
RCT: Randomized controlled trial
SAE: Serious adverse event
t1: Baseline assessment
t2: Post-intervention assessment
t3: Follow-up assessment
TAP: Test Battery of Attentional Performance
TMT: Trail making test
VLMT: Verbal learning and memory test
