# Supplementary material for: Evaluation of a memory and psychomotor training for cancer patients with cancer-related cognitive impairment: a study protocol for a prospective randomized controlled single-center trial in Germany
Source: Trials. 2025 Oct 27;26:441. doi: 10.1186/s13063-025-09211-z (PMC12560426; doi:10.1186/s13063-025-09211-z)
Supplement: Supplementary file 1 — Supplementary Material 1. [file 13063_2025_9211_MOESM1_ESM.docx]

**Supplementary material: Assessment of exclusion criteria**

Exclusion criteria were first assessed using a standardized checklist based on the electronic patient file. Eligible patients were subsequently contacted for telephone screening, during which a standardized interview guideline was applied.

A lack of German language abilities or language disorders:

1. The electronic patient file is reviewed for documentation of language disorders or notes indicating that communication is only possible through third parties, such as an interpreter or family member.

2. During telephone screening, if the patient is unable to understand the study explanation due to language barriers and communication is only possible through a third party, the participant is excluded.

Severe cognitive impairment such as dementia, brain tumors, central nervous system cancer, immobility or non-resilience of the patient, and a palliative state:

- The electronic patient file is reviewed.

Furthermore, eligible patients must have access to a terminal device with Internet access:

- This criterion is assessed during telephone screening.
